# Serum Vitamin D Level Is Unchanged in Equine Asthma

**DOI:** 10.3390/ani14223310

**Published:** 2024-11-18

**Authors:** Sophie Mainguy-Seers, Susan J. Holcombe, Jean-Pierre Lavoie

**Affiliations:** 1 Department of Clinical Sciences, Faculty of Veterinary Medicine, Université de Montréal, Saint-Hyacinthe, QC J2S 2M2, Canada; sophie.mainguy-seers@umontreal.ca; 2 Department of Large Animal Clinical Sciences, College of Veterinary Medicine, Michigan State University, East Lansing, MI 48823, USA

**Keywords:** asthma, calcitriol, heaves, inflammatory airway disease, pulmonary resistance, 25(OH)D

## Abstract

Vitamin D deficiency is associated with asthma development and severity of symptoms in humans, but whether the same occurs in horses is unknown. We aimed to determine whether the serum vitamin D levels differ in horses with asthma compared to control animals and, secondarily, to explore clinical, respiratory, and environmental parameters associated with its concentration in equids in a retrospective cross-sectional study. The total serum vitamin D (25(OH)D) was measured by radioimmunoassay in 45 serum samples from the Equine Respiratory Tissue Biobank (15 control animals, 14 horses with mild or moderate asthma (MEA), and 16 horses with severe asthma (SEA)). Descriptive clinical and environmental parameters, bronchoalveolar lavage fluid cytology, and lung function data were extracted. There was no difference in serum 25(OH)D levels between healthy controls, horses with MEA, and horses with SEA (respectively, means of 57.9 ± 11.6, 55.6 ± 20.0, and 64.6 ± 14.5 nmol/L; *p* = 0.3), suggesting that this micronutrient does not play a major role in equine asthma pathophysiology.

## Simple Summary

Equine asthma is a common inflammatory disease that decreases athletic ability, and in its severe form, negatively impacts the welfare of horses. Although genetics and environmental antigenic exposure are the main triggers of the disease, other physiological factors could influence its occurrence and severity. In humans, vitamin D deficiency is a risk factor for the development of asthma, possibly via modulation of the immune system by this micronutrient. Therefore, we aimed to determine whether vitamin D levels differ in horses affected by mild–moderate asthma, severe asthma, and healthy control horses. We observed that serum vitamin D levels were similar in all three groups, but that several physiological parameters were associated with its concentration.

## 1. Introduction

Equine asthma is a heterogeneous inflammatory disease primarily modulated by environmental conditions and genetics [1]. Other physiological factors affect its prevalence and severity, including aging [1,2], body condition score [3], and sex [2]. Yet, much remains to be uncovered on the pathophysiology and risk factors of this highly prevalent disease before targeted therapy can be implemented.

Vitamin D (25(OH)D) is a lipophilic micronutrient essential to calcium–phosphorus homeostasis and skeletal maintenance, with its main actions being to promote intestinal absorption and renal reabsorption of calcium [4,5]. In herbivores, vitamin D intake is influenced by the ingestion of ergocalciferol (vitamin D_2_) produced from ergosterols in plants following irradiation, from the skin’s production of cholecalciferol (vitamin D_3_), and from dietary supplementation [6]. Exposure of the skin to ultraviolet sunlight leads to the formation of cholecalciferol (vitamin D_3_) from 7-dehydrocholesterol. Cholecalciferol is then converted to 25-hydroxycholecalciferol in the liver, and, finally, the active form of vitamin D_3_, 1,25-dihydroxycholecalciferol (1,25(OH)_2_D_3_, also named calcitriol), is formed in the proximal tubules of the kidneys in response to parathyroid hormone stimulation [7]. Calcitriol is present in minimal concentrations in circulation and has a short half-life; therefore, total 25(OH)D is the preferred metabolite to assess an individual’s global vitamin D status [8].

This steroid hormone acts as an immunomodulator as its ligation with its ubiquitous nuclear receptor can regulate the expression of more than a hundred genes and influence several biological functions [4,9]. The mechanisms associated with the modulation of the immune system by calcitriol include the suppression of lymphocyte proliferation [10,11] and the enhanced production of regulatory T cells [12]. Consequently, vitamin D deficiency is a risk factor for asthma and other inflammatory diseases in humans [13]. In human asthma, vitamin D insufficiency has been linked to poor symptom control [14], lower lung function [15,16], and suboptimal response to corticosteroid therapy [17]. However, the lower vitamin D levels could also be consequential rather than causal in asthma, as patients perhaps spend less time outdoors due to their illness. Furthermore, the evidence for any beneficial effects of vitamin D administration in asthma is conflicting [14,18,19]. These inconsistent results might arise from the heterogeneous nature of the disease, with perhaps specific asthma endotypes being more responsive to its supplementation [16]. Particularly, the beneficial effects of vitamin D might originate from its mitigation of the Th-17 immune response [16,17], an inflammatory phenotype implicated in some subsets of asthma in humans [20] and in horses [21,22,23]. Therefore, the primary objective of this study was to determine if serum vitamin D levels differed between asthmatic and healthy horses, and, secondarily, to explore physiological factors associated with its serum concentration in horses. The hypothesis was that the serum vitamin D levels would be lower in horses affected by asthma.

## 2. Materials and Methods

### 2.1. Ethical Considerations

In this retrospective cross-sectional study, serum samples from the Equine Respiratory Tissue Biobank collected as part of other research projects were studied. Those projects were conducted in accordance with the guidelines of the Canadian Council on Animal Care and the protocols were approved by the Ethics Committee of the Université de Montréal (#Rech-1647, #Rech-1801, #Rech-1324 and #Rech-1716). For client-owned animals, the owners signed an informed consent form to store samples for research purposes.

### 2.2. Animals and Diagnosis Criteria

This study included 45 horses (15 control animals, 14 horses with mild–moderate asthma (MEA), and 16 horses with severe equine asthma (SEA)). Horses with SEA were all part of a research herd, and their diagnosis was based on a history of chronic and recurrent episodes of labored breathing, combined with the objective assessment of airway obstruction (pulmonary resistance >1 cm H_2_O/L/s measured with standard lung mechanics) and airway neutrophilia (>25% on bronchoalveolar lavage fluid (BALF) cytology on modified Wright–Giemsa stain) when stabled and fed hay. Horses with MEA were selected among animals referred to the Centre Hospitalier Universitaire Vétérinaire (CHUV) of the Université de Montréal. The diagnosis of MEA was based on the presence of compatible clinical signs (cough, nasal discharge, and/or exercise intolerance), detection of airway inflammation (≥5% neutrophils, ≥2% mast cells, and/or ≥1% eosinophils) [24], and the exclusion of other medical causes [25]. Control animals were owned by the Université de Montréal (*n* = 11, teaching herd), or presented to the CHUV (*n* = 4) for non-respiratory elective procedures. These horses were deemed healthy based on the lack of respiratory signs and normal physical examination. All horses were living in stables, with various turnout times, except for two horses with MEA living at pasture. Data gathered for each horse included age, weight, sex, breed, coat color, date of sampling, and average hours of daily sunlight during the month of sampling (climate-data.org, accessed in December 2023). The lung function and BALF data were collected as part of other research projects and have been previously published [23,26,27,28].

### 2.3. Serum Vitamin D Measurement

Blood was collected in plain vacutainer tubes (BD Biosciences, Mississauga, ON, Canada). The blood was left to clot for 20 min, centrifuged at 900× *g* for 10 min at room temperature, and then serum was collected and stored at −80 °C [29,30] until batch analysis. The total concentration of 25(OH)D (the sum of 25-hydroxyvitamin D_2_ and 25-hydroxyvitamin D_3_) in serum samples was measured in singlicate by radioimmunoassay using a radioiodinated (125I labeled) tracer (DiaSorin technique performed by Heartland Assays, Ames, IA, USA) [31]. The intra- and inter-assay coefficients of variation were, respectively, 4.4% and 11.9%.

### 2.4. Statistical Analyses

Normality was assessed with Shapiro–Wilk tests and visual analysis of a QQ-plot. The effect of the disease status, breed categories, and horse provenance on serum vitamin D levels, as well as the comparisons of clinical data within the 3 disease groups (age, weight, BALF cytology), were analyzed with one-way ANOVA with Bonferroni tests for multiple comparisons, or when non-normally distributed, with Kruskal–Wallis and Dunn’s tests. The breeds were separated into 4 categories (Light breeds (Thoroughbreds and Standardbreds), heavy breeds (Haflingers, Canadians, Belgians), Quarter Horse related breeds (Quarter Horses, Paint Horses, Appaloosas), and Warmbloods). Unpaired *t*-tests, or Mann–Whitney tests if the data were not normally distributed, were used to explore the effect of dichotomous variables (sex and coat color) on vitamin D levels, and to compare lung function data between control and SEA horses. Coat colors were dichotomously separated based on the extension loci (dominance; bay or black, or recessive; chestnut) to assess the role of the type of melanin (respectively, eumelanin and phaeomelanin) [32]. Other types of coat colors were not analyzed as they were too sparse, and the coat color was not recorded in 11 horses. Associations between serum 25(OH)D and continuous data (age, weight, average hours of daily sunlight during the month of sampling, airway neutrophilia, and lung function) were explored with Pearson or Spearman correlations. Data are described with the mean and standard deviation. Statistical analyses were performed with GraphPad Prism version 10.1.2 for Windows (GraphPad Software, San Diego, CA, USA) with *p* < 0.05 considered significant.

## 3. Results

### 3.1. Clinical Information

The control group included six Standardbreds, four Quarter Horses or Quarter Horse crossbreds, three Thoroughbreds, and two Haflingers; the MEA group included five Quarter Horses, two Paint Horses, three Warmbloods, two Canadian Horses, one Belgian, and one Thoroughbred; and the SEA group included eight Quarter Horses or Quarter Horse crossbreds, three Standardbreds, two Paint Horses, one Belgian, one Appaloosa and one Canadian crossbred. The weight of the animals did not differ between groups (controls, MEA horses, and SEA horses weighed, respectively, 473 ± 48 kg (weight missing for one horse), 521 ± 130 kg, and 508 ± 70 kg (weight missing for one horse)). The age of the horses was significantly different between groups (ANOVA *p* = 0.0001), with horses with MEA (6.9 ± 5.1 years, *p* = 0.0001) and control horses (9.3 ± 5.2 years, *p* = 0.004) being younger than horses with severe asthma (16 ± 6.1 years). As standard lung function is used in the research facility but not in the clinical setting, lung function data at the time of sampling was available for all horses with SEA, in 10/15 control animals, and in none of the horses with MEA. All horses with SEA had pulmonary resistances consistent with clinical exacerbation (>1 cm H_2_O/L/s, mean of 2.9 ± 1.2 cm H_2_O/L/s), while control horses had normal lung function (mean resistance of 0.6 ± 0.2 cm H_2_O/L/s, *p* < 0.0001). The BALF cytology results were available on all horses. The degree of airway neutrophilia was statistically different between groups (ANOVA *p* < 0.0001); it was higher in severe asthmatic horses (39.9 ± 25.6%) compared to controls (6.5 ± 3.3%, *p* < 0.0001) and to horses with MEA (19.8 ± 21.2%, *p* = 0.02). The percentages of mast cells in the BALF cytology were also different between groups (ANOVA *p* < 0.0001); it was higher in horses with MEA (3.2 ± 1.9%) compared to controls (0.5 ± 0.7%, *p* < 0.0001) and to horses with SEA (0.6 ± 0.6%, *p* < 0.0001).

### 3.2. Serum Vitamin D Levels

**Figure 1:**
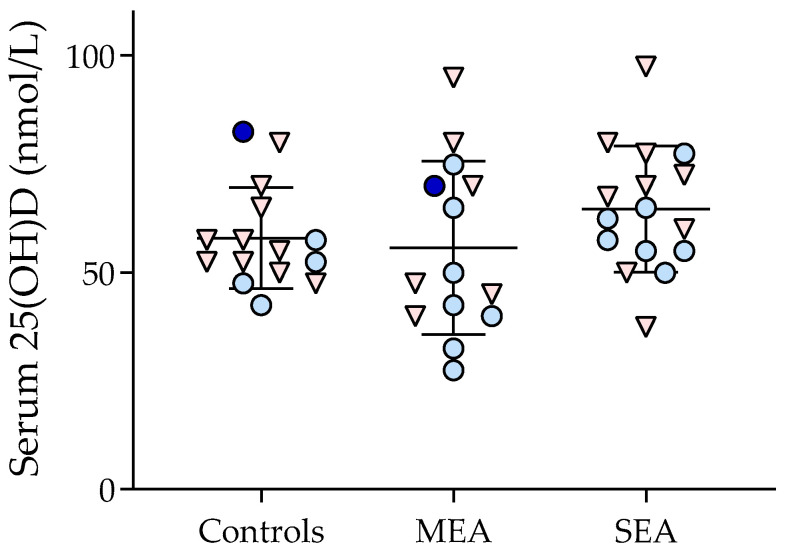
Serum 25(OH)D levels in control and asthmatic horses in a scatter dot plot with the middle line representing the mean and the lower and upper lines representing the standard deviation. Mares, geldings, and stallions are illustrated with pink triangles, light blue circles, and dark blue circles, respectively. MEA = mild–moderate asthma. SEA = severe equine asthma. 25(OH)D = total vitamin D.

The serum vitamin D level was not different between disease groups (57.9 ± 11.6, 55.6 ± 20.0, and 64.6 ± 14.5 nmol/L), respectively, for control horses and horses with MEA and SEA, *p* = 0.3 (Figure 1). Because the horses affected by SEA were older, the analysis was repeated while removing all horses older than 15 years old, and it did not influence the result (no group difference in vitamin D levels). As the disease status did not affect vitamin D levels, all horses were considered together for the exploration of factors associated with its serum concentration.

The serum vitamin D levels were higher in mares compared to geldings (*p* = 0.04, respectively, 63.0 ± 16.0 and 53.0 ± 13.3 nmol/L) and were higher in horses carrying the extension loci genotype (dominance (bay or black); 64.4 ± 12.4 nmol/L, *n* = 18) compared to horses that expressed the recessive trait (chestnut; 52.5 ± 14.5 nmol/L, *n* = 8, *p* = 0.02). The serum vitamin D levels did not vary based on the provenance of the horses (research herd, teaching herd, or client-owned), the breed categories, or the weight.

**Figure 2:**
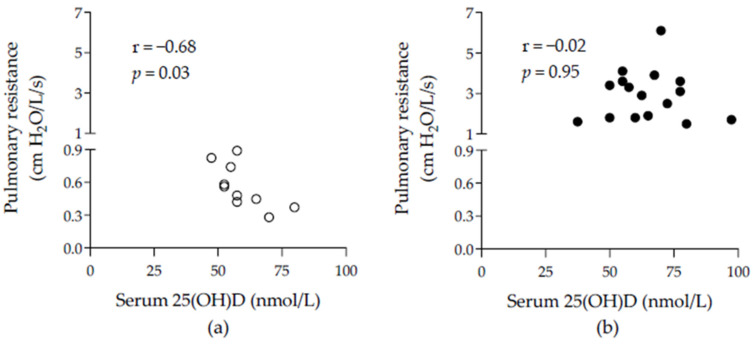
Correlation between serum 25(OH)D and pulmonary resistance. (**a**) in healthy horses (blank circles) and (**b**) in horses with severe equine asthma (filled circles). 25(OH)D = total vitamin D.

There was a positive correlation between vitamin D levels and the daily hours of sunlight during the month of sampling (Spearman r = 0.34, *p* = 0.02), and between vitamin D levels and age (Pearson r = 0.47, *p* = 0.001). There was a negative correlation between pulmonary resistance and serum vitamin D levels, but only within control horses (Pearson r = −0.68, *p* = 0.03, Figure 2). Of note, this association could not be assessed in the MEA horses as lung function was unavailable in this group. There was no correlation between vitamin D levels and airway neutrophilia, whether the groups were considered separately or together.

## 4. Discussion

Contrary to the initial hypothesis, the findings of this retrospective cross-sectional study indicate that there is no difference in serum vitamin D levels between healthy controls and horses with asthma, regardless of severity. However, preliminary exploration showed that sex, coat color, age, and daily hours of sunlight during the month of sampling might be related to vitamin D status. Importantly, a negative correlation between circulating 25(OH)D and pulmonary resistance was detected in the small cohort of healthy controls, indicating that this vitamin might influence airway physiology.

The overall mean serum vitamin D level of 60 nmol/L in the current study is similar to other reports using radioimmunoassay [33] or enzyme immunoassay [29], but is higher than reported when using high-performance liquid chromatography (≈11.3–37.5 nmol/L) [6,34,35], highlighting the large difference related to measurement methods [36]. Nevertheless, all equine studies clearly show that horses have serum vitamin D concentrations far lower than most animal species and humans, in which values below 50 nmol/L are associated with negative outcomes, including rickets and osteomalacia [13]. These low 25(OH)D values, combined with the high circulatory and urinary calcium naturally observed in horses, question the biological importance of this hormone in the equine species [33,37], and perhaps have contributed to the absence of a relation to asthma status in horses in the current study compared to humans.

The correlation between lower airway resistance and higher serum 25(OH)D levels in healthy horses is a novel finding in this species but is similar to the association of vitamin D deficiency to decreasing lung function both in healthy [38] and asthmatic humans [15]. This improved airflow could be related to the effects of vitamin D on structural cells of the airways [39], as it reduces airway smooth muscle proliferation and its production of metalloproteinase-9 [40], two processes that contribute to bronchial wall thickening. This correlation could have been masked in the severe asthmatic horses because more potent factors likely contribute to bronchospasms during disease exacerbation, such as the degree of antigenic exposure and environmental conditions. As the association was based on clinical data available from only ten control animals, this finding should, however, be interpreted cautiously and confirmed in a larger cohort. Additional investigations in large cohorts of horses would be required to determine if the serum vitamin D level influences airway resistance in SEA horses during periods of clinical remission and in MEA, as data on lung function was not collected in this latter group in the current study. Whether the association between vitamin D levels and lung function in healthy animals is reflected in athletic performance also deserves further investigation.

Although there was a large overlap between the sexes, the mares had higher serum vitamin D levels than the geldings. These results differ from a previous study where no sex difference was observed [33], while other authors reported an effect of the sex, but did not specify whether mares or geldings had higher levels [41]. Of note, the two stallions included in the current cohort had among the highest 25(OH)D concentrations (70 and 82.5 nmol/L), but more animals are needed to determine if this latter observation is a coincidence. In humans, the serum 25(OH)D levels are generally lower in women than in men [42], a phenomenon possibly related to the positive association between testosterone and vitamin D levels [43,44,45]. Furthermore, the lower levels in geldings are in line with the low circulatory vitamin D levels in men suffering from hypogonadism [45]. Castration does not, however, affect the 1α,25(OH)_2_D_3_ vitamin D concentrations up to 6 months after the surgery in horses [46], but a delayed effect of the procedure was not excluded in that study. As androgens possess bronchodilator properties [47], it would be of interest to evaluate the possible interrelation between the reported association between testosterone and vitamin D, and the correlation between vitamin D and improved lung function in the current study.

The increased serum concentration of total 25(OH)D with age observed in the current study is similar to results reported for 25(OH)D_3_ in Thoroughbreds from Thailand [29]. However, other authors did not observe an effect of age on total and 1,25(OH)_2_D [33] and no difference was obtained for 1α,25(OH)_2_D_3_ between post-pubertal (≈2 years) and adult horses [46]. While these discrepancies highlight the lack of understanding of the vitamin D physiology in the equine species, a potential explanation resides in the type of metabolite analyzed. Nonetheless, the association between 25(OH)D levels and age in the current study is noteworthy, as horses with SEA were unsurprisingly older. However, repeating the analyses while removing all horses older than 15 years did not modify the results, suggesting that the age difference between groups was unlikely to have influenced the outcome.

The weak correlation between vitamin D levels and daily sunlight observed in the current study is consistent with the seasonal effect reported by others [6,30,41,48], and illustrates that vitamin D status might differ considerably based on latitudinal location. However, as the total vitamin D levels (25(OH)D_2_ and 25(OH)D_3_) were measured, it remains undetermined whether the correlation was caused by skin sunlight exposure or by higher consumption of vitamin D_2_ in the diet from pasture, from which the content is higher during days with extended sunshine [30]. Combined, the very low serum vitamin D_3_ levels [30], the lack of effect of blanketing on vitamin D levels [41], and the absence of vitamin D_3_ synthesis by equine skin after ultraviolet B radiation in vitro [49] suggest that endogenous skin production is not the main source of total vitamin D in horses. However, this hypothesis conflicts with the unexpected finding that horses carrying the extension loci trait for eumelanin skin coloration had higher serum vitamin D levels. In humans, people with dark skin color are at increasing risk of vitamin D deficiency [50], a phenomenon generally thought to be related to the increased amount of skin melanin [51]. Therefore, the intriguing finding that horses with phaeomelanin skin coloration have lower circulatory 25(OH)D needs confirmation in a larger cohort, as is the contribution of the skin on vitamin D biosynthesis in the equine species. The differentiation of (25(OH)D_2_ and 25(OH)D_3_), with a technique such as high-performance liquid chromatography, would be required to further the understanding of vitamin D metabolism in horses.

The main limitations of the current study are related to its retrospective nature, as the information collected did not include the diet and use of supplements, the turnout duration, the exact timing of blood collection during the day, the exercise level of the horses, or body condition score, all of which could influence serum vitamin D levels [6,30,48,52]. Furthermore, the SEA group was likely more homogenous than the control or MEA groups, although this limitation was mitigated by the collection of samples over 3 years with varying management in the research herd. Overall, this study showed that asthmatic horses were not more likely to suffer from vitamin D deficiency. However, whether low levels could contribute to other clinical parameters related to vitamin D levels in humans, such as the initial onset of the disease, corticosteroid resistance, or the development of clinical exacerbation, were not examined [17,53,54]. Furthermore, a power calculation was not performed a priori, given the lack of existing data on the equine species in asthma. From the results gathered in the current study, approximately 60 horses in each group would have been necessary to observe a significant difference between the groups with a power of 80%, bearing in mind that vitamin D levels were numerically higher in SEA, and not lower as hypothesized. This high number of horses suggests that if a difference exists, its clinical relevance would require scrutiny. Another limitation of this study is that some control horses had airway neutrophil percentages greater than 5%, and, as most controls were teaching mares, exercise intolerance could have been missed in some of those horses. Even though asthma is not diagnosed based solely on the presence of airway inflammation, and healthy horses can develop transient airway neutrophilia when exposed to hay [55], this limitation could have occulted the role of airway inflammation on vitamin D status.

## 5. Conclusions

In conclusion, serum vitamin D levels did not differ in asthmatic horses compared to control animals. The effect of sex, age, daily duration of sunlight, coat color, and the association between improved airway resistance and vitamin D levels in healthy horses require confirmation in larger cohorts of horses.

## Data Availability

The data that supports the findings of this study is openly available in the UdeM Dataverse repository at https://doi.org/10.5683/SP3/M5VEO6.
